# Predictive Factors for Structural and Functional Outcomes of 25-Gauge Pars Plana Vitrectomy (PPV) for the Removal of Posterior Intraocular Foreign Bodies (IOFBs): A Retrospective Data Analysis From North India

**DOI:** 10.7759/cureus.69686

**Published:** 2024-09-18

**Authors:** Ashish Markan, Mohit Dogra, Ramandeep Singh, Vishali Gupta, Basavaraj Tigari

**Affiliations:** 1 Ophthalmology, Post Graduate Institute of Medical Education and Research, Chandigarh, IND

**Keywords:** 25-gauge, intraocular foreign body, pars plana vitrectomy, predictive factors, surgical outcomes

## Abstract

Purpose: To identify predictive factors for structural and functional outcomes of 25-gauge pars plana vitrectomy (25G PPV) for removal of posterior segment intra-ocular foreign body (IOFB).

Methodology: A retrospective data analysis was performed for patients undergoing 25G PPV for removal of posterior segment IOFB between August 2019 to June 2021. Necessary demographic details and data regarding pre-operative ophthalmic examination were recorded. Similarly, intraoperative surgical details were recorded. Postoperative outcome measures included final best corrected visual acuity (BCVA), retinal status, and epiretinal membrane formation at the last follow-up visit. A univariate analysis was applied to find the association of various independent variables with functional and structural outcomes.

Results: Thirty-nine patients were included in the study, with 37 males and two females. The mean age of the study group was 30.5+10.8 years. The most common zone of open globe injury was zone 1. Most of the impactions of IOFB were seen outside the macular area. Preoperative BCVA was 2.23+0.58 logarithm of the Minimum Angle of Resolution (logMAR), which improved significantly to 1.01+0.53 in the postoperative period (p-value <0.001). Anatomical success was achieved in 92.3% of patients at one year follow-up. The presence of impacted IOFB, associated endophthalmitis and IOFB >4mm were associated with poor visual outcomes (univariate analysis; p-value <0.05). None of the factors affected the anatomical success rates.

Conclusion: The presence of impacted IOFB, associated endophthalmitis, and large IOFB (>4mm) were associated with poor visual outcomes with 25G PPV for removal of IOFB.

## Introduction

Intraocular foreign bodies (IOFBs) refer to objects that penetrate the eye and become lodged within its structures, posing a significant threat to visual function. IOFBs can vary in composition and size, ranging from metallic fragments and glass shards to organic matter [1]. The management of IOFBs typically involves surgical removal to minimize the risk of complications and preserve visual acuity [2]. However, the success of IOFB removal procedures can depend on several predictive factors that influence surgical outcomes.

The posterior segment location of the IOFB within the eye can significantly impact surgical success due to associated retinal detachment, vitreous hemorrhage, or proliferative vitreoretinopathy (PVR) [3]. Similarly, impacted IOFBs may require additional surgical maneuvers, such as tissue dissection or the use of intraocular forceps, which can increase the risk of complications and potentially affect the surgical success [4]. Other factors like timing of surgery, associated retinal detachment, endophthalmitis, size of IOFB, presenting visual acuity are also known to affect surgical outcomes [5-9]. Small gauge vitrectomies, like 23-gauge (23G) or 25-gauge (25G) are associated with shorter operating time, faster recovery, and improved patient comfort during the post-operative period. We, in this study, looked at various pre-operative and intra-operative factors and studied their association with surgical success of 25-gauge PPV for removal of posterior IOFB.

## Materials and methods

This retrospective study aimed to investigate the predictive factors associated with structural and functional outcomes of 25-gauge pars plana vitrectomy (25G PPV) for the removal of posterior intraocular foreign bodies (IOFBs). The study period was two years (August 2019 to June 2021). The study adhered to the principles outlined in the Declaration of Helsinki.

All patients underwent 25G PPV under local anesthesia using the Constellation vitrectomy system (Alcon, Geneva, Switzerland). Surgeries were performed by three different surgeons (AM, BT, MD) with equal surgical experience. Lensectomy was performed in all cases of traumatic cataract and in cases where IOFB> 5mm, to avoid large scleral wound. IOFB < 5mm were extracted via enlargement of the sclerotomy site. Primary intraocular lens implantation was not performed in any of the cases requiring lensectomy. The decision to put an encircling element, route of IOFB removal, and choice of intraocular tamponade was decided by the operating surgeon.

Preoperative, intraoperative, and postoperative data were collected from the medical records of patients who underwent 25G PPV for posterior IOFB removal. The following preoperative data were recorded: age, gender, best-corrected visual acuity (BCVA), zone of open globe injury, nature and size of IOFB, presence of associated retinal detachment (RD) or endophthalmitis, presence of cataract, and timing of surgery (<two weeks or >two weeks). Zone of open globe injury was defined as per Ocular trauma classification group [6]. Zone 1 injuries associated with involvement of visual axis was noted.

Intraoperative surgical details included whether an encircling element was placed, whether a lensectomy was performed, the route of IOFB removal (limbal or scleral), and whether any tamponade was used at the end of the surgery. Lensectomy was performed in all cases of traumatic cataract and in cases where IOFB> 5mm, to avoid large scleral wound.

Impacted foreign body was defined as penetration of foreign body through the retinal tissue into the ocular coats. The zone of impaction in the retina and choroidal tissue was divided into Zone 1, Zone 2, and Zone 3. Zone 1 was defined as IOFB impacted within the macula, Zone 2 was defined as impaction present outside the macular area but posterior to the equator, and Zone 3 was defined as impaction present anterior to the equator.

Postoperative outcomes were studied at one year of follow-up. These included BCVA, retinal status, and the presence or absence of epiretinal membrane (ERM). Functional success was defined as BCVA better than 6/60, while anatomical success was defined as no retinal detachment (RD) involving the macular area at the time of the last follow-up [7].

Univariate analysis was performed to assess the association between the preoperative and intraoperative factors mentioned above and the structural and functional outcomes of the surgery. Descriptive statistics were used to summarize the demographic and clinical characteristics of the study population. Continuous variables were reported as means with standard deviations or medians with interquartile ranges, while categorical variables were presented as frequencies and percentages. Statistical significance was determined using appropriate tests, such as the chi-square test or Fisher's exact test for categorical variables and t-tests or Mann-Whitney U tests for continuous variables. A p-value of less than 0.05 was considered statistically significant.

## Results

A total of 39 participants were included in the study. The mean age of the patients was 30.02+10.54 years. The gender distribution showed a significant majority of male participants (94.9%, n=37), with only a small percentage being female (5.1%, n=2). The majority of the injuries were workplace-related (79.4%, n=31), followed by home and domestic accidents (15.38%, n=6) and bystander injuries (5.12%, n=2).

The mean best corrected visual acuity (BCVA) was 2.23+0.58 log MAR. Zone 1 injuries were the most prevalent, accounting for 59% (n=23) of cases, followed by Zone 2 injuries (30.8%,n=12) and Zone 3 injuries (10.2%, n=4). More than 60% (n=14) of Zone 1 injuries involved central 2mm of the cornea, thus affecting the visual axis.

Regarding the nature of foreign bodies, metallic objects were identified in the majority of cases (89.7%, n=35), while non-metallic foreign bodies were present in a smaller proportion (10.3%, n=4). The timing of surgery revealed that a significant majority of participants underwent surgery within two weeks of injury (87.2%, n=34), while the remaining participants underwent surgery after two weeks (12.8%, n=5). The delay in surgical intervention in these eyes was primarily due to corneal edema and poor media quality. Post-traumatic cataract was seen in 71.7% (n=28) of patients, thus hampering the visualization. Lensectomy was performed in 82% (n=32) of patients, either because of cataracts or to remove large IOFB (>5mm) through the limbal route. 

Impaction of IOFB into the ocular coats was encountered in 66.67% (n=26) of eyes. Out of these 26 impacted foreign bodies, one-third of patients (n=12) required extensive Chori retinectomy. Perfluorocarbon liquid (PFCL) was used in all our cases to prevent any inadvertent damage to the fovea during IOFB manipulation. Primary internal limiting membrane (ILM) peeling was not performed in any of our cases. Intraocular tamponade was used in all cases of retinal detachment, cases requiring extensive Chori retinectomy, and the presence of large retinal breaks. The choice of tamponade, whether oil or gas, was decided by the operating surgeon. Ten cases (25.6%) required silicone oil tamponade, 13 cases (33.3%) required gas tamponade, and 16 cases required no long-term intraocular tamponade.

All eyes with associated endophthalmitis were operated within 48 hours of presentation. Out of 12 eyes with endophthalmitis, only one had associated retinal detachment. Intravitreal ceftazidime (2.25 mg/0.1ml) and vancomycin (1mg/0.1ml) were empirically injected at the end of surgery. Table 1 highlights the detailed demographic profile and clinical characteristics of patients included in the study.

**Table 1:** Demographic and injury related characteristics of study participants (N=39) The data has been represented as N (%). IOFB: Intraocular foreign bodies; FB: Foreign body.

Characteristics	N (%)
Gender	
Female	2 (5.1%)
Male	37 (94.9%)
Zone of injury	
1	23 (59%)
2	12 (30.8%)
3	4 (10.2%)
Zone of impaction	
1 (inside the arcades)	6 (15.4%)
2 (from arcades to the equator)	10 (25.6%)
3 (anterior to equator)	10 (25.6%)
Impacted IOFB	
Yes	26 (66.7%)
No	13 (33.3%)
Nature of FB	
Metallic	35 (89.7%)
Non-metallic	4 (10.3%)
Timing of surgery	
<2 weeks	34 (87.2%)
>2 weeks	5 (12.8%)
Encirclage element (240 no.) used	
Yes	23 (59%)
No	16 (41%)
Associated retinal detachment	
Yes	8 (20.5%)
No	31 (79.5%)
Associated endophthalmitis	
Yes	12 (30.8%)
No	27 (69.2%)
Associated traumatic cataract	
Yes	28 (71.8%)
No	11 (28.2%)
Lensectomy performed	
Yes	32 (82.1%)
No	7 (17.9%)
Route of extraction	
Limbal	33 (84.6%)
Scleral	6 (15.4%)
Size of foreign body	
Small (<4mm)	28 (71.79%)
Large (>4mm)	11 (28.20%)
Tamponade	
Oil	10 (25.6%)
Gas	13 (33.3%)
None	16 (41.1%)

At one year of follow-up, the mean post-operative BCVA improved to 1.01+0.53 logarithm of the Minimum Angle of Resolution (logMAR) (p-value <0.001). The retina was attached in 82% (n=32) of patients, and epiretinal membrane (ERM) formation occurred in 23% (n=9) of patients.

Out of 39 patients, 18% (n=7) developed retinal detachment during the follow-up period. Amongst these, three patients underwent a repeat retinal detachment surgery. Other secondary interventions involved: secondary IOL implantation (12 patients), silicon oil removal (five patients), vitreous lavage (two patients), and ERM removal (2 patients).

Results from the univariate analysis showed no association between age, gender, zone of injury), nature of IOFB, timing of surgery, associated pre-operative retinal detachment, associated cataract, lensectomy performed, route of IOFB removal, and presence or absence of tamponade with postoperative BCVA. A detailed analysis of various independent factors with post-operative BCVA has been shown in Table 2.

**Table 2:** Association between postop BCVA and independent variables among study participants (N=39) IOFB: Intraocular foreign bodies; RD: Retinal detachment; ERM: Epiretinal membrane; 3d: Three-dimensional; BCVA: Best-corrected visual acuity; OR: Odds ratio. The data has been represented as N (%). P-value was considered significant if p<0.05.

Characteristics	Post op BCVA		OR, 95%CI	P value	Univariate analysis	
	Log max <1 (Good Vision) N (%)	Log max >1 (poor vision) N (%)			Odds ratio, 95% CI	P value
Age						
<30	12 (50)	12 (50)	0.87,0.24-3.18	1.00	1.14, 0.31-4.16	0.83
>30	8 (53.3)	7 (46.7)				
Gender						
Female	1 (50)	1 (50)	0.94, 0.05-16.30	1.00	1.05, 0.06-18.17	0.97
Male	19 (51.4)	18 (48.6)				
Zone of injury						
Within Zone 1	19 (54.3)	16 (45.7)	3.5, 0.33-37.68	0.34	0.28, 0.02-2.97	0.29
Outside Zone 1	1 (25)	3 (75)				
Zone of impaction						
Within Zone 1	2 (33.3)	4 (66.7)	0.41, 0.06-2.59	0.40	2.40, 0.38-14.96	0.34
Outside Zone 1	18 (54.5)	15 (45.5)				
Impacted IOFB						
Yes	10 (38.5)	16 (61.5)	0.18, 0.04-0.85	0.04	5.33, 1.17-24.21	0.03
No	10 (76.9)	3 (23.1)				
Nature of IOFB						
Metallic	18 (51.4)	17 48.6)	1.06, 0.13-8.38	1.00	0.94, 0.11-7.47	0.95
Non-metallic	2 (50)	2 (50)				
Timing of surgery						
<2 weeks	17 (50)	17 (50)	0.66, 0.09-4.51	1.00	1.50, 0.22-10.14	0.67
>2 weeks	3 (60)	2 (40)				
Encirclage element						
Yes	13 (56.5)	10 (43.5)	1.67, 0.46-6.05	0.52	0.59, 0.16-2.16	0.43
No	7 (43.8)	9 (56.3)				
Associated RD						
Yes	3 (37.5)	5 (62.5)	0.49, 0.10-2.43	0.45	2.02, 0.41-9.99	0.38
No	17 (54.8)	14 (45.2)				
Associated endophthalmitis						
Yes	3 (25)	9 (75)	0.19, 0.04-0.89	0.04		
No	17 (63.2)	10 (37)			5.10, 1.11-23.37	0.03
Associated cataract						
Yes	13 (46.4)	15 (53.6)	0.49, 0.11-2.08	0.48	2.01, 0.48-8.48	0.33
No	7 (63.5)	4 (36.4))				
Lensectomy performed						
Yes	15 (46.9)	17 (53.1)	0.35, 0.05-2.09	0.40	2.83, 0.47-16.81	0.25
No	5 (71.4)	2 (28.6))				
Route of IOFB removal						
Limbal	15 (45.5)	18 (54.5)	0.16, 0.02-1.58	0.18	6.00, 0.63-57.13	0.11
Scleral	5 (83.3)	1 (16.7)				
Size of IOFB						
<4mm	18 (64.3)	10 (35.7)	8.10, 1.45-45.06	0.01	0.12, 0.02-0.68	0.01
>4mm	2 (18.2)	9 (81.8)				
Tamponade used						
Yes	9 (39.1)	14 (60.9)	0.29, 0.07-1.12	0.10	3.42, 0.88-13.18	0.07
No	11 (68.8)	5 (31.3)				
Longest follow-up						
<3	13 (52)	12 (48)	1.08, 0.29-4.01	1.00	0.92, 0.24-3.41	0.90
>3	7 (50)	7 (50)				
Retina status post-op						
Attached	19 (59.4)	13(40.6)	8.76, 0.94-81.67	0.04	0.114, 0.01-1.06	0.05
Detached	1 (14.3)	6 (85.7)				
ERM post-op						
Yes	4 (44.4)	5 (55.6)	0.70, 0.15-3.13	0.71	1.42, 0.31-6.38	0.64
No	16 (53.3)	14 (46.7)				
Viewing system						
Standard operating microscope	5 (31.3)	11 (68.8)	0.24, 0.06-0.94	0.05	4.12, 1.05-16.09	0.04
3D Heads Up Display	15 (65.2)	8 (34.8)				

On the other hand, impacted IOFB, associated endophthalmitis, and size of IOFB had a significant association with final BCVA. The presence of an impacted foreign body showed a statistically significant association with postoperative BCVA (p-value=0.04, 95% CI=1.17-24.21), indicating that participants without an impacted foreign body had better functional outcomes. Similarly, participants without endophthalmitis had better functional outcomes following their surgery (p-value=0.03, 95% CI=1.11-23.37). The size of IOFB demonstrated a statistically significant association with postoperative BCVA (p=0.01, 95% CI=0.02-0.68). Participants with smaller IOFBs (<4mm) had better functional success compared to those with larger IOFBs (>4mm).

None of the pre-operative or intra-operative factors had any association with structural outcomes, ie retinal status and ERM formation. A detailed analysis of various independent factors with retinal status and epiretinal membrane formation in the postoperative period has been shown in Table 3.

**Table 3:** Association between retina attachment/detachment and independent variables among study participants (N=39) IOFB: Intraocular foreign bodies; FB: Foreign body; RD: Retinal detachment; 3D: Three-dimensional; OR: Odds ratio. The data has been represented as N (%). P-value was considered significant if p<0.05.

Characteristics	Retina Attached N (%)	Retina detached N (%)	OR, 95%CI	P value	Univariate analysis	
					OR, 95%CI	P value
Age						
<30	20 (83.3)	4(16.7)	1.25, 0.23-6.56	1.00	0.91, 0.17-4.77	0.91
>30	12 (80)	3 (20)				
Gender						
Female	2 (100)	0		1.00	NA	0.99
Male	30 (81.1)	7 (18.9)				
Zone of injury						
Within Zone 1	29 (82.9)	6 (17.1)	1.61, ]0.14-18.26	0.56	0.62, 0.05-7.03	0.70
Outside Zone 1	3 (75)	1 (25)				
Zone of impaction						
Within Zone 1	5 (83.3)	1 (16.7)	1.11, 0[.10-11.33	1.00	0.90, 0.08-9.17	0.92
Outside Zone 1	27 (81.8)	6 (18.2)				
Impacted IOFB						
Yes	19 (73.1)	7 (26.9)		0.07	NA	0.99
No	13 (100)	0				
Nature of FB						
Metallic	28 (80)	7 (20)		1.00	NA	0.99
Non Metallic	4 (100)	0				
Timing of surgery						
<2 weeks	28 (82.4)	6 (17.6)	1.16, 0.11-12.38	1.00	0.85,0.08-9.09	0.89
>2 weeks	4 (80)	1 (20)				
Encirclage Element						
Yes	20 (87)	3 (13)	2.22, 0.42-11.67	0.42	0.45, 0.08-2.36	0.34
No	12 (75)	4 (25)				
Associated RD						
Yes	7 (87.5)	1 (12.5)	1.68, 0.17-16.37	1.00	0.59,0.06-5.80	0.65
No	25 (80.6)	6 (19.4)				
Associated endophthalmitis						
Yes	8 (66.7)	4 (33.3)	0.25, 0.04-1.36	0.17	4.00, 0.73-21.83	0.11
No	24 (88.9)	3 (11.1)				
Associated cataract						
Yes	24 (85.7)	4 (14.3)	2.25, 0.41-12.28	0.37	0.44,0.08-2.42	0.34
No	8 (72.7)	3 (27.3)				
Lensectomy performed						
Yes	26 (81.3)	6 (18.8)	0.72, 0.07-7.17	1.00	1.38, 0.13-13.75	0.78
No	6 (85.7)	1 (14.3)				
Route of IOFB removal						
Limbal	26 (78.8)	7 (21.2)		0.56	NA	0.99
Scleral	6 (100)	0				
Size of IOFB						
<4mm	22 (78.6)	6 (21.4)	0.36, 0.03-3.46	0.64	2.72, 0.28-25.74	0.38
>4mm	10 (90.9)	1 (9.1)				
Tamponade used						
Given	19 (82.6)	4 (17.4)	1.09, 0.205.73	1.00	0.91, 0.17-4.77	0.91
Not given	13 (81.3)	3 (18.8)				
Viewing system						
Standard operating microscope	12 (75)	4 (25)	0.45, 0.08-2.36	0.41	0.69, 0.13-3.69	0.67
3D Heads Up Display	20 (87)	3 (13)				

A detailed analysis of various independent factors with epiretinal membrane formation in the postoperative period has been shown in Table 4.

**Table 4:** Association between ERM post op and independent variables among study participants (N=39) IOFB: Intraocular foreign bodies; FB: Foreign body; RD: Retinal detachment; ERM: Epiretinal membrane; OR: Odds ratio; 3D: Three-dimensional. The data has been represented as N (%). P-value was considered significant if p<0.05.

Characteristics	Yes N (%)	No N (%)	OR, 95%CI	P value	Univariate analysis	
					OR, 95%CI	P value
Age						
<30	7 (29.2)	17 (70.8)	2.67, 0.47-15.08	0.43	0.32,0.05-1.83	0.20
>30	2 (13.3)	13 (86.7)				
Gender						
Female	0	2 (100)	NA	0.29	NA	0.99
Male	9 (24.3)	28 (75.7)				
Zone of injury						
Within Zone 1	8 (22.9)	27 (77.1)	0.88, 0.08-9.76	1.00	1.12, 0.10-12.36	0.92
Outside Zone 1	1 (25)	3 (75)				
Zone of impaction						
Within Zone 1	5 (83.3)	1 (16.7)	1.11, 0.10-11.33	1.00	0.22, 0.03-1.38	0.10
Outside Zone 1	27 (81.8)	6 (18.2)				
Impacted IOFB						
Yes	6 (23.1)	20 (76.9)	1.00, 0.20-4.85	1.00	1.00, 0.20-4.85	1.0
No	3 (23.1)	10 (76.9)				
Nature of FB						
Metallic	9 (25.7)	26 (74.3)	NA	0.55	NA	0.99
Non Metallic	0	4 (100)				
Timing of surgery						
<2 weeks	8 (23.5)	26(76.5)	1.23,0.12-12.65	1.00	0.81, 0.07-8.35	0.86
>2 weeks	1 (20)	4 (80)				
Encirclage Element						
Yes	8 (34.8)	15 (65.2)	8.00, 0.88-72.09	0.06	0.12, 0.01-1.12	0.06
No	1 (6.3)	15 (93.8)				
Associated RD						
Yes	1 (12.5)	7 (87.5)	0.41, 0.04-3.87	0.65	2.43, 0.25-22.97	0.43
No	8 (25.8)	23 (74.2)				
Associated endophthalmitis						
Yes	5 (41.7)	7 (58.3)	4.1, 0.86-19.61	0.10	0.23, 0.05-1.16	0.07
No	4 (14.8)	23 (85.2)				
Associated cataract						
Yes	5 (17.9)	23 (82.1)	0.38, 0.08-1.81	0.23	2.62, 0.55-12.55	0.22
No	4 (36.4)	7 (63.6)				
Lensectomy performed						
Yes	8 (25)	24 (75)	2.00, 0.20-19.22	1.00	0.50, 0.05-4.80	0.54
No	1 (14.3)	6 (85.7)				
Route of IOFB removal						
Limbal	9 (27.3)	24 (72.7)		0.30	NA	0.99
Scleral	0	6 (100)				
Size of IOFB						
<4mm	6 (21.4)	22 (78.6)	0.72, 0.14-3.62	0.69	1.37, 0.27-6.84	0.69
>4mm	3 (27.3)	8 (72.7)				
Tamponade used						
Yes	6 (26.1)	17 (73.9)	1.52, 0.32-7.29	0.71	0.65, 0.13-3.12	0.59
No	3 (18.8)	13 (81.3)				
Longest follow-up						
<3 months	4 (16)	21 (84)	0.34, 0.07-1.58	0.23	2.91, 0.63-13.45	0.17
>3 months	5 (35.7)	9 (64.3)				
Retina status						
Attached	8 (25)	24 (75)	2.00, 0.20-19.22	1.00	0.50, 0.05-4.80	0.54
Detached	1 (14.3)	6 (85.7)				
Viewing system						
Standard operating microscope	3 (18.8)	13 (81.3)	0.65, 0.13-3.12	0.71	NA	NA
3D Heads Up Display	6 (26.1)	17 (73.9)				

## Discussion

The index study conducted a comprehensive analysis of predictive factors influencing anatomical and functional success in the context of 25G PPV for IOFB removal. Notably, the presence of impacted IOFB, associated endophthalmitis, and large IOFB (>4mm) emerged as significant determinants of unfavorable functional outcomes.

Our study showed that predominantly most of our patients were male with a mean age of 30.5+10.8 years. Our results are consistent with previous studies, which have shown that IOFB is a serious problem in the young working-age population, especially males [5,8]. One possible explanation for this could be linked to gender-related behavior and a higher level of male participation in high-risk work activities. The majority of the IOFBs in our study were metallic, which is similar to what has been reported in other studies [5,8].

Previous studies have shown Zone 3 injuries to be associated with poor visual outcomes [9]. This is primarily due to an increased risk of retinal and vitreous incarceration, retinal damage, and vitreous hemorrhage in Zone 3 injuries. Our study showed no association between the zone of injury and final visual acuity. The majority of our patients had a Zone 1 injury and involved the visual axis. Approximately 45% patients with zone 1 injury had poor visual outcome. This could primarily be due to the involvement of the visual axis and association with traumatic cataracts in Zone 1 injuries.

In our study, impacted foreign bodies exhibited a clear correlation with unfavorable functional outcomes. This aligns with findings from previous research that also identified a similar association [4,10]. The underlying reasons for this link can be attributed to the extensive damage caused to ocular tissues, the heightened risk of subretinal and choroidal hemorrhage, the development of extensive epiretinal membranes, increased surgical complexities, and the necessity for extensive chorio-retinectomy in cases involving impacted foreign bodies.

Our study findings strongly correlated associated endophthalmitis with unfavorable functional outcomes, which aligns consistently with prior research [11]. Factors such as endophthalmitis-related damage to the retina and optic nerve are well-known to restrict optimal visual recovery. Contrary to this, several other studies did not find any association of endophthalmitis with visual outcomes [5,8,12].

Additionally, our study revealed that larger IOFBs (>4mm) had an adverse impact on visual outcomes, indicating them as poor prognostic factors. This can primarily be attributed to the extensive damage inflicted on ocular tissues by larger foreign bodies and the surgical challenges encountered during their removal [12].

Our study do not advocate for the use of prophylactic encircling elements in IOFB management. The data on the effectiveness of prophylactic buckles in this context remains limited. Our findings are in line with the study conducted by Peng et al. [11]. However, the study by Azad et al. recommended the use of prophylactic buckles for all IOFB cases, despite showing no statistical difference between the two groups [13]. It's worth noting that their study utilized a 20-gauge vitrectomy technique. In today's era, with advancements in instrumentation and the widespread adoption of small gauge vitrectomies, the necessity of employing a prophylactic scleral buckle has diminished.

The method of IOFB extraction did not influence structural and functional outcomes in our study. While the choice of extraction route may vary based on the surgeon's preference, caution is recommended when dealing with large IOFBs, as removing them through pars plana incisions could elevate the risk of retinal or vitreous incarceration, as well as iatrogenic retinal breaks. Majority of IOFB extraction was done through limbal route. As majority of our patients required lensectomy, it was easier to remove the IOFB through the limbal route.

In our study, the timing of surgery had no bearing on structural and functional outcomes. The optimal timing of PPV for IOFB retrieval remains a topic of debate, with conflicting results in the literature. Studies suggest a higher risk of endophthalmitis and formation of proliferative vitreoretinopathy in cases where the removal is delayed [14-16]. Interestingly, the study by Colyer et al. found no elevated risk of endophthalmitis even with delayed removal [10].

While some studies advocate for early intraocular foreign body (IOFB) removal within the first 24-48 hours, we hold the perspective that performing IOFB removal during or shortly after globe repair presents surgical challenges due to the inherent instability of corneal or scleral wounds in this early phase. Our stance emphasizes the importance of conducting IOFB removal a few days after globe repair, allowing for greater wound stability and optimizing surgical outcomes. In our practice, the majority of patients underwent IOFB removal within the initial two weeks following globe repair.

As a standard protocol, all our patients receive prophylactic antibiotics during the globe repair procedure. The contention surrounding the administration of intravitreal antibiotics during globe repair revolves around its potential to prevent or delay the onset of clinically significant endophthalmitis [17]. Notably, our study's findings indicate a predominant prevalence of metallic IOFBs, which have been associated with a diminished risk of endophthalmitis when compared to organic IOFBs [18]. These two factors collectively provide a basis for the consideration of a delayed approach, allowing a time window of one to two weeks before IOFB removal.

All patients underwent a sequential approach, with open globe injury (OGI) repair done as a primary surgery. This was followed by PPV surgery for removal of IOFB. The mean delay in second stage surgery was 7.2+1.2 days. This delay serves a dual purpose. Firstly, it contributes to the stabilization of the wound sites, ensuring a more controlled and secure surgical environment. Secondly, deferring IOFB removal aids in the natural progression of spontaneous posterior vitreous detachment, a phenomenon that holds the potential to enhance the efficiency and success of subsequent pars plana vitrectomy (PPV) procedures. The development of spontaneous posterior vitreous detachment before surgery contributes to the smoother execution of the PPV, thus enhancing overall surgical outcomes.

To sum up, our approach advocates for a delayed IOFB removal strategy following globe repair, ideally within the first one to two weeks. This approach is supported by the administration of prophylactic antibiotics, the reduced risk of endophthalmitis associated with metallic IOFBs, and the benefits of wound stabilization and spontaneous posterior vitreous detachment development, all of which collectively contribute to improved surgical results.

In contrast to previous research suggesting an association between pre-existing retinal detachment and reduced functional gains, our study did not find such a link [5,12]. This discrepancy may be attributed to our emphasis on early intervention (<2 weeks) in the majority of our patients. Additionally, difference could also happen because of a smaller sample size in our study population and warrants a long-term study with a larger sample size.

Post-operatively ERM was seen in 23% (n=9) of patients of open globe injury at 11 months of follow-up. This incidence is in accordance with what has been described previously. The ERM after PPV is usually visually insignificant. In our study population, majority of the patients developed a grade 0 ERM, with a normal foveal contour. Only two patients underwent ERM peeling, due to formation of macular pucker.

While our study offers valuable and comprehensive preoperative, intraoperative, and postoperative information regarding visual outcomes following surgical procedures for posterior segment IOFBs, it is essential to acknowledge its potential limitations. These limitations include the relatively small sample size and the retrospective, nonrandomized design. Despite these drawbacks, our research contributes important insights into the field of IOFB-related surgeries.

## Conclusions

In conclusion, our index study conducted an extensive analysis of predictive factors influencing anatomical and functional success in 25G PPV for IOFB removal. Though 25 gauge does not confer additional advantage over large gauge vitrectomies as far as structural and functional outcomes are concerned, but is associated with shorter operating time, faster recovery and improved patient comfort during the post-operative period. 

Our study shed light on the impact of various factors on visual outcomes, providing valuable insights for improving IOFB management strategies. The presence of impacted IOFBs, associated endophthalmitis, and large IOFBs emerged as significant determinants of unfavorable functional outcomes in our study.
